# Honey bees overwintering in a southern climate: longitudinal effects of nutrition and queen age on colony-level molecular physiology and performance

**DOI:** 10.1038/s41598-018-28732-z

**Published:** 2018-07-11

**Authors:** Vincent A. Ricigliano, Brendon M. Mott, Amy S. Floyd, Duan C. Copeland, Mark J. Carroll, Kirk E. Anderson

**Affiliations:** 1 USDA-ARS Carl Hayden Bee Research Center, Tucson, AZ 85719 USA; 2 Department of Entomology and Center for Insect Science, University of Arizona, Tucson, AZ 85721 USA; 3 Department of Microbiology, School of Animal & Comparative Biomedical Sciences; University of Arizona, Tucson, AZ 85721 USA

## Abstract

Honey bee colony nutritional ecology relies on the acquisition and assimilation of floral resources across a landscape with changing forage conditions. Here, we examined the impact of nutrition and queen age on colony health across extended periods of reduced forage in a southern climate. We measured conventional hive metrics as well as colony-level gene expression of eight immune-related genes and three recently identified homologs of *vitellogenin* (*vg*), a storage glycolipoprotein central to colony nutritional state, immunity, oxidative stress resistance and life span regulation. Across three apiary sites, concurrent longitudinal changes in colony-level gene expression and nutritional state reflected the production of diutinus (winter) bees physiologically altered for long-term nutrient storage. Brood production by young queens was significantly greater than that of old queens, and was augmented by feeding colonies supplemental pollen. Expression analyses of recently identified *vg* homologs (*vg-like-A*, -*B*, and *-C*) revealed distinct patterns that correlated with colony performance, phenology, and immune-related gene transcript levels. Our findings provide new insights into dynamics underlying managed colony performance on a large scale. Colony-level, molecular physiological profiling is a promising approach to effectively identify factors influencing honey bee health in future landscape and nutrition studies.

## Introduction

Honey bee (*Apis mellifera*) colony loss occurs primarily during the winter regardless of climate. Although overwintering in temperate regions has been well studied^1–4^, relatively little is known of the behavioral, nutritional, and molecular physiological responses from honey bees overwintering in climates of the southern United States. A sizable proportion of US colonies overwinter in warm climates throughout the southern U.S. including southern California, Texas and Florida. In southern climates, shorter and warmer winters can allow for longer growth seasons, but unsustainable brood rearing and foraging activity can also deplete scarce resources during extended periods of forage dearth accompanied by abrupt bouts of colder temperatures. Colony losses are often attributed to poor nutrition, queen failure, compromised immune function, increases in parasites/pathogens, or a combination of these factors^5–9^.

The western honey bee is adapted to survive seasonal differences in temperature and forage dearth by storing simple sugars in the hive and more complex nutrient stores within the bodies of long-lived workers. This cohort of workers transforms physiologically into a nutrient storage caste referred to as “diutinus” bees^10^. Following extended forage dearth, the nutrients stored in diutinus bees are used to synthesize food for a new cohort of brood. This colony-level nutritional economy is reliant on the production and conservation of internal storage molecules, predominantly *vitellogenin*, a nutritionally-regulated protein that is highly expressed during the fall months leading up to winter^11–13^. Diutinus bees accumulate excess levels of *vitellogenin*, which increases their life-span and improves their tolerance to starvation and oxidative stress^10,14^.

To compensate for suboptimal forage conditions and high-density apiaries where natural pollen is scarce, colonies are typically fed artificial (pollen substitute) diets^15–18^. Some studies have found that artificial diets are inferior to diets containing natural pollen^19–21^. Compared to natural pollen, artificial diets feature atypical phytochemical and enzymatic profiles that can influence honey bee physiology beyond their primary nutritional roles^22–28^. One approach to improving honey bee nutrition might incorporate a fraction of natural pollen or components found in a natural diet of pollen and nectar into supplemental feeding regimes. Pollen consumption by workers results in molecular signaling central to honey bee metabolism and aging that is partially mediated by fermentation products produced by hindgut bacteria^29–31^. The expression of immune genes is also affected by microbiome dynamics and pollen consumption, suggesting that prebiotic properties of pollen could also modulate immunity^12,32–34^.

A common goal of honey bee research is to effectively distill colony-level factors into simplified metrics that accurately reflect colony performance under various landscape conditions and commercial management settings. For example, intensively cultivated landscapes and migratory management conditions are significantly associated with reduced colony performance and increased levels of oxidative stress^6,9,35–39^. Forage availability and nutrient balance are fundamental to honey bee processes such as brood production, immune function and overwintering survival^12,34,40–44^. Additionally, poor nutrition is correlated with a variety of sub-lethal effects including altered immune response and increased susceptibility to pathogens and environmental xenobiotics^13,45–48^. Nutritional landscape fluctuations and seasonal forage dearth are also associated with increased queen loss^7,49,50^ highlighting queen age or queen “events” in longitudinal studies^51–54^. Colonies with young queens also have larger adult populations and increased productivity compared to colonies with older queens^50,55–57^.

Queen quality and nutrition play an important role in honey bee colony performance and health. As far as we are aware, no large-scale studies have explored relevant management practices during the critical months leading up to winter in a warm southern climate. Here we present a longitudinal study of honey bee colony performance and physiological status in the Imperial Valley of California during a seven month period from July through January. The Imperial Valley represents an agriculturally intensive, warm winter environment with low rainfall and low diversity and abundance of natural forage that necessitates supplemental feeding. We manipulated two factors, queen age and supplemental feed composition, across three distinct apiary sites. We monitored traditional hive metrics of brood production, pollen stores, and pathogen levels as well as nutritional and immune gene expression in pooled worker samples. We tested whether prophylactic queen replacement and supplemental pollen feeding are viable approaches to improve colony performance in a commercial management context. We also hypothesized that a pooled sampling approach would overcome individual variation to more closely reflect colony-level physiological status and establish connections between performance, gene expression and phenology. To further assess the utility of molecular biomarkers at the colony level, we analyzed the expression of recently reported *vg-like* gene homologs implicated in physiology related to life-span regulation and oxidative stress response^58^.

## Results

### Effects of queen age and supplemental pollen feeding

**Figure 1:**
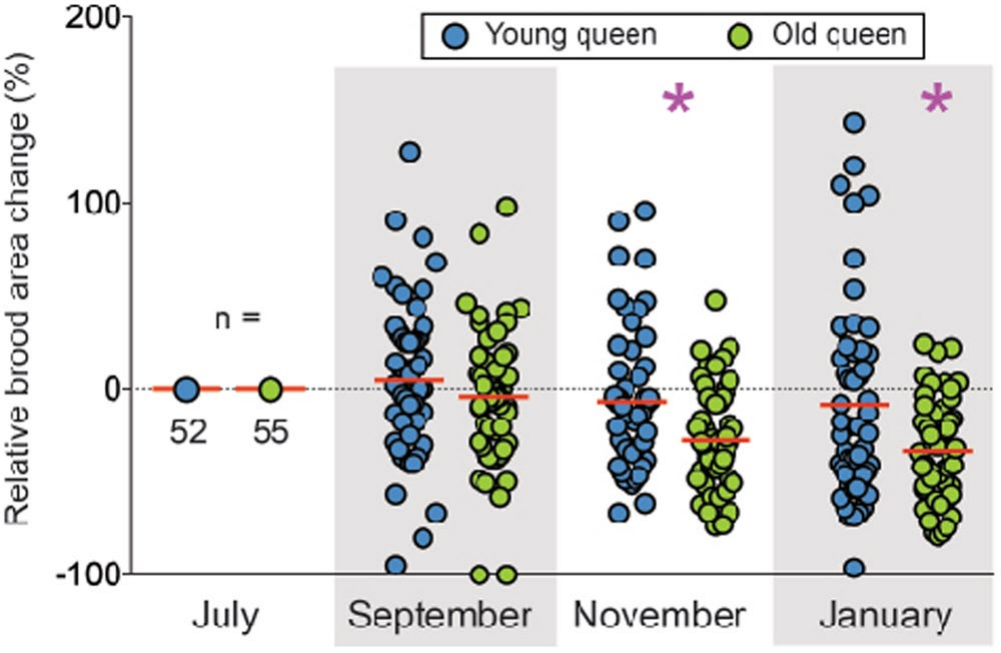
Colonies that harbored young queens outperformed colonies that harbored old queens with respect to total brood production. Colonies with young queens produced 24% and 25% more brood in November and January, respectively. Values represent the percent change in total brood area relative to July. Red horizontal lines indicate the mean. Asterisks indicate statistically significant differences in November and January at α = 0.05.

We compared colonies that harbored either a young queen or old queen and were fed either artificial diet containing no pollen or artificial diet containing 25% natural pollen. Surveying 252 colonies across 3 apiaries, queen age had no discernable effect on colony survival (Supplementary Fig. S1). However, surviving colonies with young queens produced significantly more brood overall compared to colonies with old queens (F_1,105_ = 10.06, P = 0.002; Fig. 1). Brood production by young queens in November and January was 24% and 25% greater than that of old queens, respectively. Supplemental feeding with natural pollen led to a 16.3% increase in brood production compared to exclusively artificial feed, but this increase was not statistically significant (*F*_1,105_ = 1.608, P = 0.208, Supplementary Fig. S2). We detected an interaction between diet and evaluation time point on brood production (*F*_2,210_ = 3.782, P = 0.024; Supplementary Fig. S2), suggesting that the efficacy of artificial feed formulations are impacted by season-dependent, nutritional landscape dynamics. Consistent with this finding, an interaction between diet and evaluation time point led to detection of increased *vg-like-A* expression in November in colonies fed pollen (P = 0.023; Supplementary Fig. S3), suggesting that pollen feeding increased the production of *vg-like-A* in diutinus bees relative to exclusively artificial feed. Besides the aforementioned effect on vg-like-A expression, feed composition did not significantly influence colony-level gene expression (data not shown).

**Figure 2:**
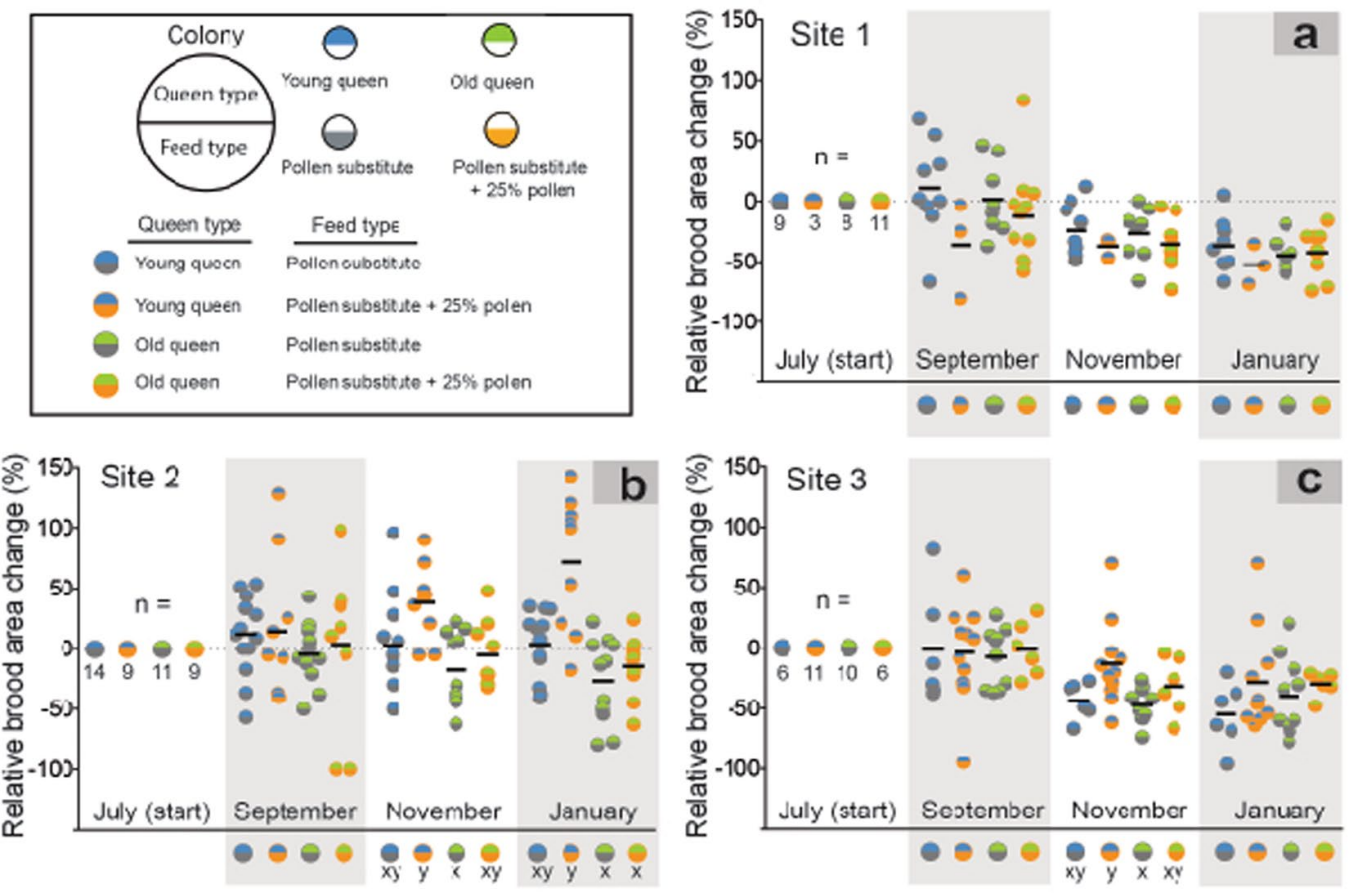
The effects of queen age and supplemental pollen feeding on colony brood production. Colonies harbored either young queens or old queens and received supplemental feed consisting of either artificial pollen substitute or the same feed containing 25% natural pollen (w/w). Values represent the percent change in brood area relative to July. Black horizontal lines indicate the mean. Within each site and evaluation period, different letters indicate statistically significant differences among treatment combinations at α = 0.05.

Treatment effects differed by site. No treatment effects were detected at site 1 (P > 0.05; Fig. 2a). At site 2 in November, young queen colonies fed pollen had significantly increased brood areas compared to old queen colonies fed no pollen (*X*^2^ = 11.316, df = 3, P = 0.010; Fig. 2b) At site 2 in January, young queen colonies fed pollen produced significantly more brood than old queen colonies fed no pollen and old queen colonies fed pollen (*X*^2^ = 17.106, df = 3, P < 0.001; Fig. 2b). At site 3 in November, young queen colonies fed pollen had significantly increased brood production compared to old queen colonies fed no pollen (*X*^2^ = 9.145, df = 3, P = 0.027; Fig. 2c).

### Expression of *vitellogenin* homologs *vg-like-A*, *-B*, and *-C*

**Figure 3:**
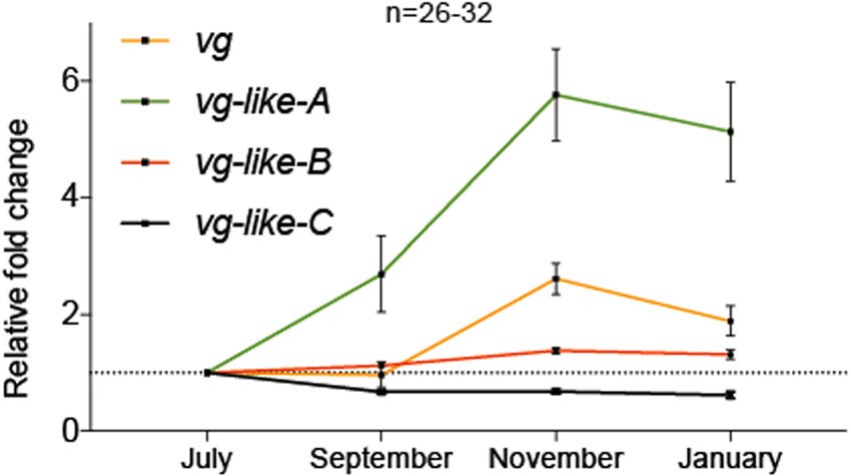
Fall through winter colony-level expression patterns of *vg-like* genes. Values represent fold changes relative to July mRNA levels and normalized to actin. Error bars represent standard error (SE).

Increased fall *vg* expression is a characteristic attribute of diutinus bee production. We tested the prediction that *vg*-like gene transcripts are expressed according to seasonal patterns of diutinus (typically overwintering) bees. Transcript levels of *vg-like-A* revealed distinct spatiotemporal expression patterns and transcript levels of *vg-like-A*, *-B*, and *-C* were all significantly influenced by evaluation time point (Supplementary Fig. S4). Compared to *vg* transcript levels, temporal expression differences in *vg-like-A* were detectable in September. In November, *vg-like-A* expression increased 5.8-fold in whereas *vg* increased only 2.6-fold, suggesting that *vg-like-A* may play an important role in colony-level physiology associated with diutinus worker production (Fig. 3).

### Immune-related gene expression

**Figure 4:**
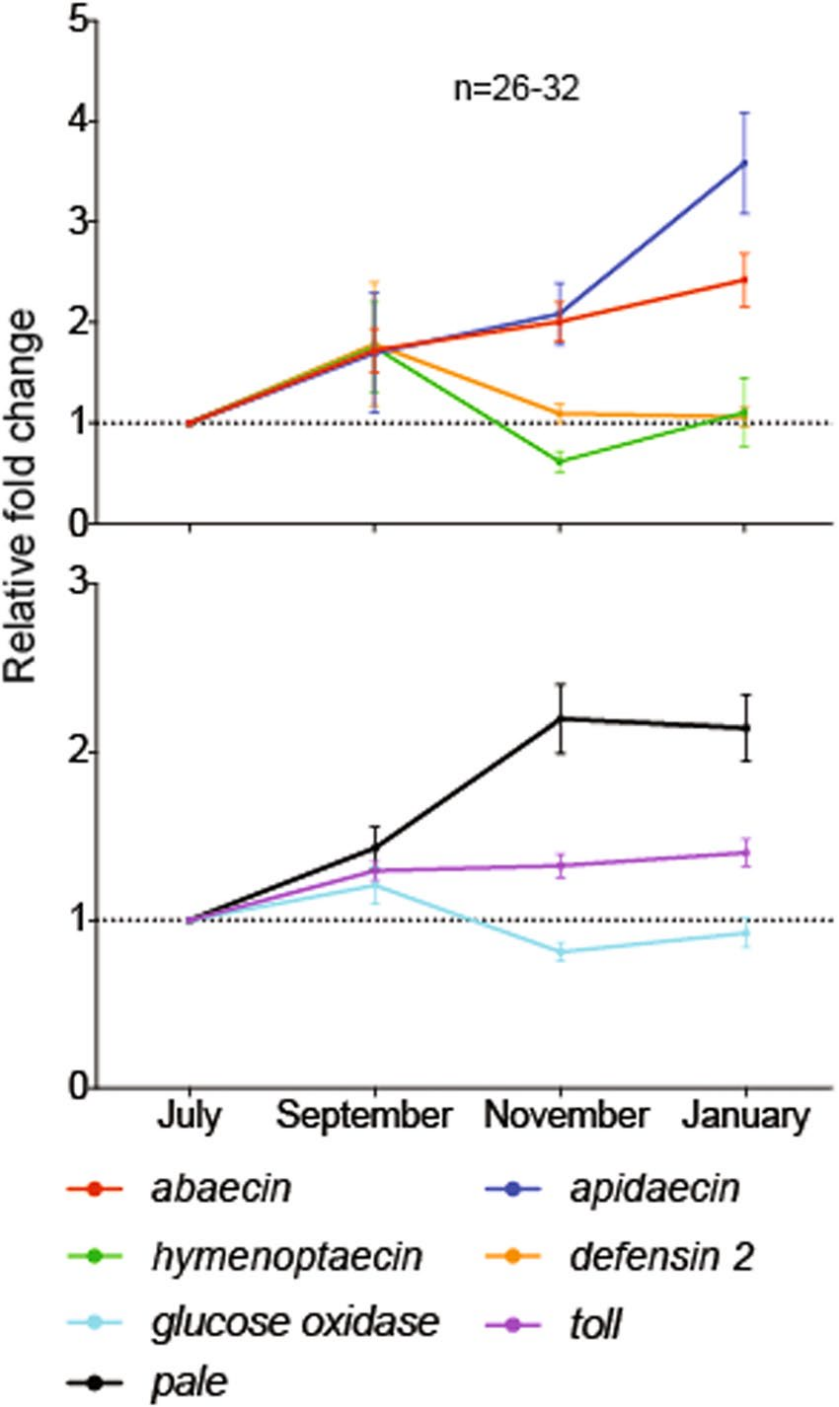
Fall through winter temporal expression patterns of immune-related genes. Values represent fold changes relative to July mRNA levels.

Colony-level expression analyses revealed significant effects of evaluation time point on transcript levels of the immune-related genes *abaecin*, *apidaecin*, *hymenoptaecin*, *defensin 2*, *glucose oxidase*, *toll*, and *pale* (Fig. 4 and Supplementary Fig. S5). Relative to July, January expression of the antimicrobial peptides *abaecin* and *apidaeicin* increased 2.4- and 3.5-fold, respectively (Fig. 4). Similarly, expression of *pale* increased approximately 2-fold and expression of *toll* increased approximately 1.4-fold (Fig. 4). Expression levels of the antimicrobial peptide *apidaecin* were significantly influenced by site (*F*_2,116_ = 3.565, P = 0.031; Supplementary Fig. S6). An interaction between evaluation time point and apiary site led to detection of significantly different transcript levels of glucose oxidase among sites (*F*_6,117_ = 4.133, P < 0.001; Supplementary Fig. S6). Expression of *pale*, a tyrosine hydroxylase involved in melanization and response to bacterial challenge, was significantly influenced by site (*F*_2,117_ = 8.748, P < 0.001; Supplementary Fig. S6).

### Effects of apiary site on hive performance and *vitellogenin* expression

**Figure 5:**
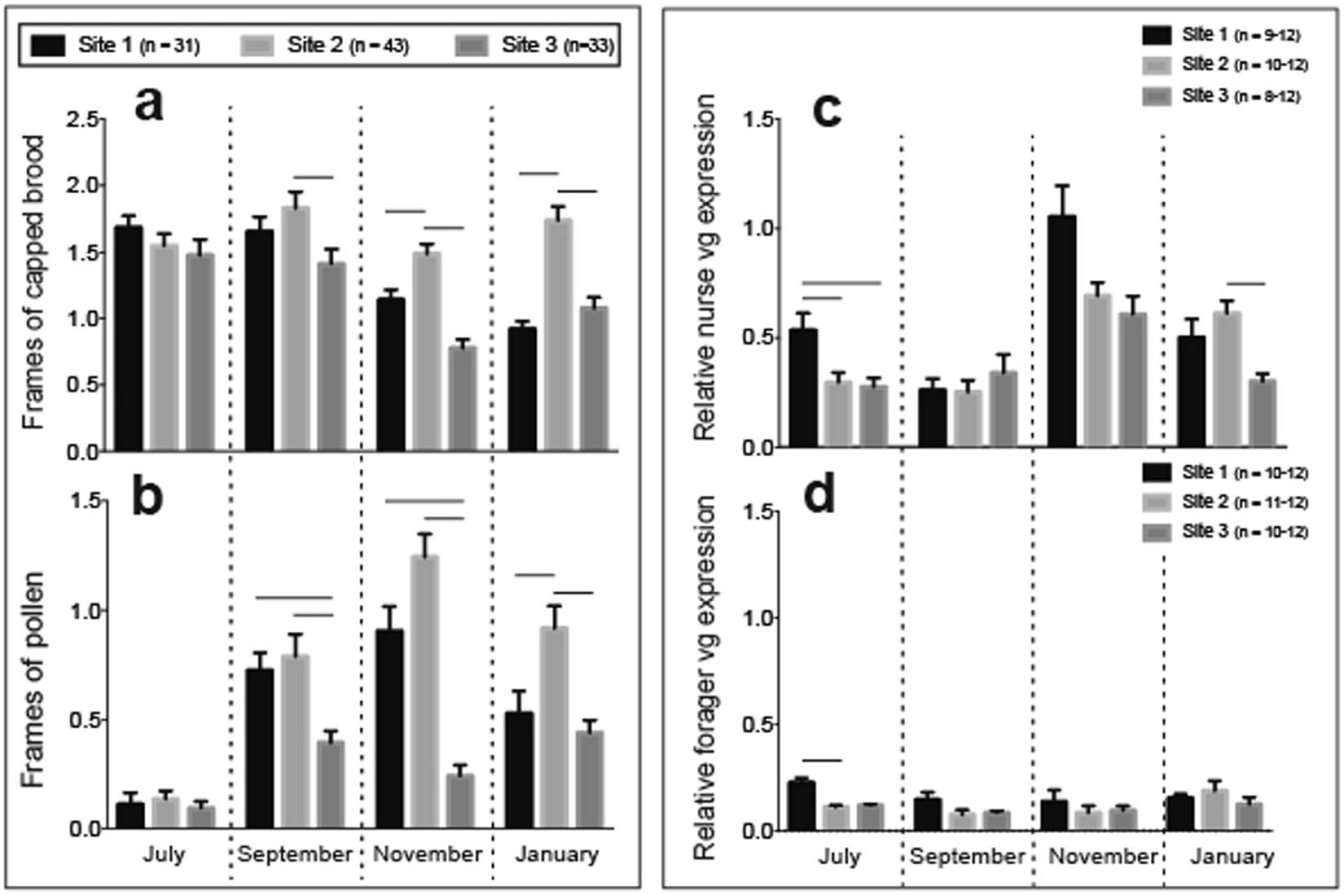
The effects of apiary site on honey bee hive performance and colony-level *vg* expression. (**a**) Average frames of capped brood per colony measured as whole frame coverage equivalents. (**b**) Average pollen accumulation measured as whole frame coverage equivalents. (**c**) Relative *vg* expression in pooled nurse bee samples collected from inside colony brood nests. (**d**) Relative *vg* expression in pooled forager bee samples collected from outside hive entrances. Error bars represent standard error (SE). Horizontal lines above columns indicate statistically significant differences at α = 0.05.

From July-January, apiary site had a marked effect on total brood production (*F*_2,104_ = 8.354, P < 0.001; Supplementary Fig. S7) and pollen storage. In September, honey bee colonies at site 2 produced significantly more capped brood than site 3 (*F*_2,104_ = 13.47, P < 0.001; Fig. 5a). In November and January, colonies at site 2 produced significantly more capped brood relative to both of the other apiary sites (Fig. 5a and Supplementary Fig. S8). In September and November, colonies at site 3 accumulated significantly less pollen than sites 1 and 2 (September: *X*^2^ = 9.941, df = 2, P = 0.007 and November: *X*^2^ = 40.975, df = 2, P < 0.001; Fig. 5b). In January, colonies at site 2 accumulated significantly more pollen than sites 1 and 3 (*X*^2^ = 17.085, df = 2, P < 0.001; Fig. 5b).

Relative *vitellogenin* (*vg*) mRNA levels were measured in separate pools of nurse and forager worker bees from a random subset of colonies at each apiary site. Pooled nurse workers had significantly higher *vg* transcript levels than pooled forager workers (Fig. 5c,d). Site had a significant effect on *vg* expression in nurses (*F*_2,116_ = 7.542, P < 0.001; Fig. 5c) and foragers (*F*_2,120_ = 4.791, *P* = 0.010; Fig. 5d). Site effects varied by evaluation time point for *Vg* expression, which could be attributed to environmental fluctuations and pre-winter colony phenology (nurses; *F*_3,116_ = 26.9, P < 0.001 and foragers; *F*_3,120_ = 3.023, *P* = 0.032).

Significant correlations were observed between total brood production, pollen stores, and nurse *vg* expression (Supplementary Fig. S9). The strength and significance of the relationships varied by time. Notably, nurse *vg* expression in September was positively correlated with pollen stores (r_sq_ = 0.609, P < 0.001) and total brood area (r_sq_ = 0.436, P = 0.018), indicating the potential of *Vg* as a molecular diagnostic that partially recapitulates standard hive metrics. July brood area was predictive of September brood area (r_sq_ = 0.485, P = 0.006), September pollen stores (r_sq_ = 0.447, P = 0.012), January brood area (r_sq_ = 0.405, P = 0.024) and January pollen stores (r_sq_ = 0.447, P = 0.012). Within-month correlations between brood area and pollen stores were identified in November (r_sq_ = 0.427, *P* = 0.021) and January (r_sq_ = 0.510, P = 0.003).

### *Varroa*, deformed wing virus, and *Nosema ceranae* levels

**Figure 6:**
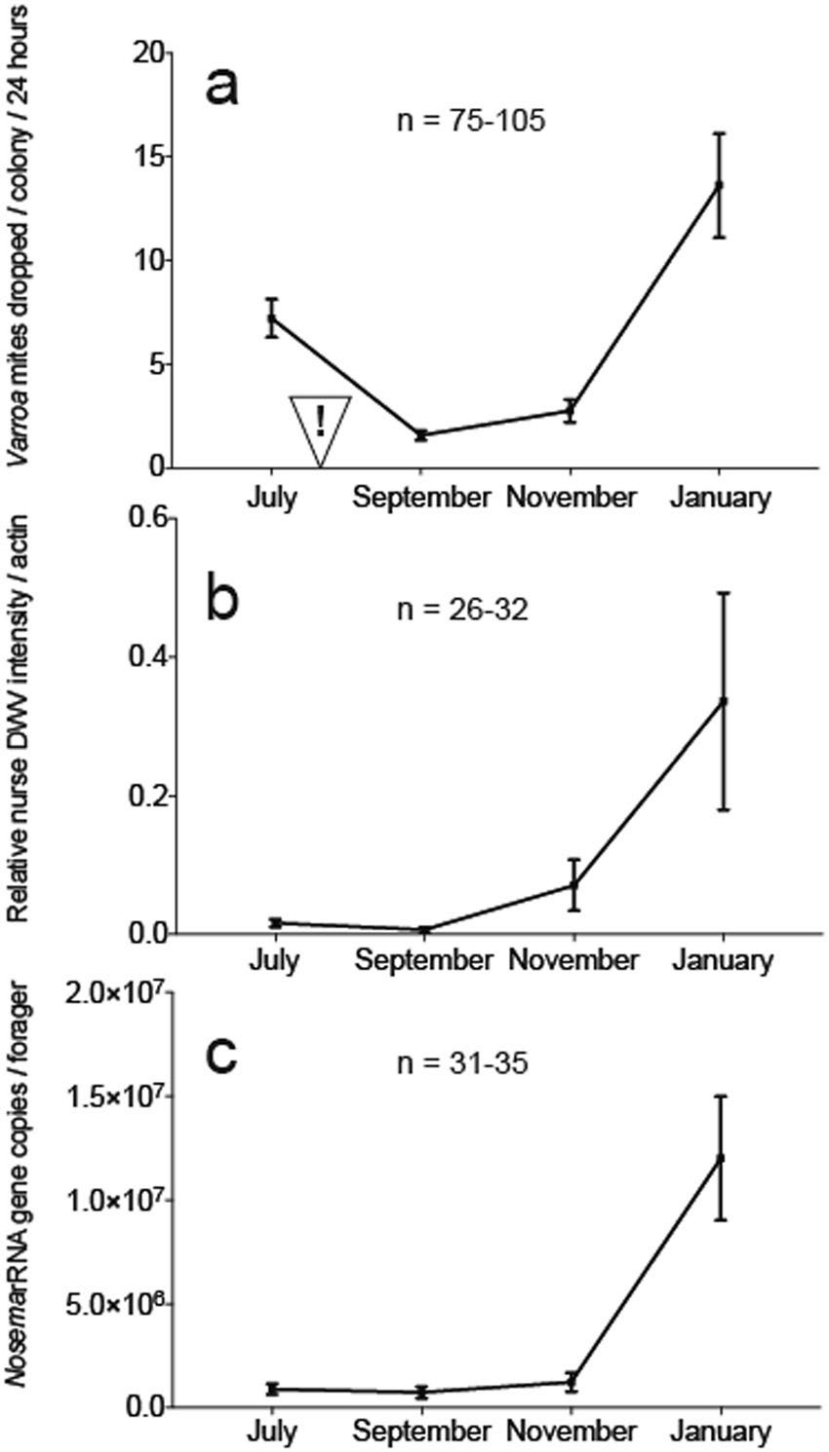
*Varroa*, deformed wing virus, and *Nosema* levels at each apiary site. (**a**) *Varroa* levels measured by passive mite drop per hive per 24 hours. A miticide treatment was applied in August and is indicated by (!) (**b**) Relative deformed wing virus levels in pooled nurse bee samples normalized to host actin and expressed on a log scale. (**c**) Absolute quantification of *Nosema* 16S rDNA (rRNA gene) copies in pooled forager samples expressed per bee. Error bars represent standard error (SE).

*Varroa*, deformed wing virus (DWV), and *Nosema* levels were monitored throughout the experiment. Significant differences in pathogen levels were observed with respect to evaluation time point. At the start of colony evaluations in July, colonies had significantly higher *Varroa* levels than in September and November (*X*^2^ = 103.369, df = 3, P < 0.001; Fig. 6a) which could be attributed to an acaricide treatment applied in late August. Following the acaricide application, *Varroa* levels were reduced 4.6-fold in September and began to increase by November although still reduced 2.6-fold from starting levels in July. Overall, deformed wing virus levels in nurse workers were significantly reduced from July to September and were highest in January (*X*^2^ = 33.593, df = 3, P < 0.001; Fig. 6b). Both nurse and forager DWV levels mirrored colony *Varroa* levels (Fig. 6b and Supplemental Fig. S10). From July-November, the prevalence of *Nosema* infection was below 10^6^ rRNA gene copies per bee in nurse and forager workers. In January, forager *Nosema* levels reached 10^7^ rRNA gene copies per bee, which was significantly higher than July-November levels (*X*^2^ = 48.330, df = 3, P < 0.001; Fig. 6c) and nearly 10-fold higher than nurse levels (Supplemental Fig. S11).

### Molecular correlations

**Figure 7:**
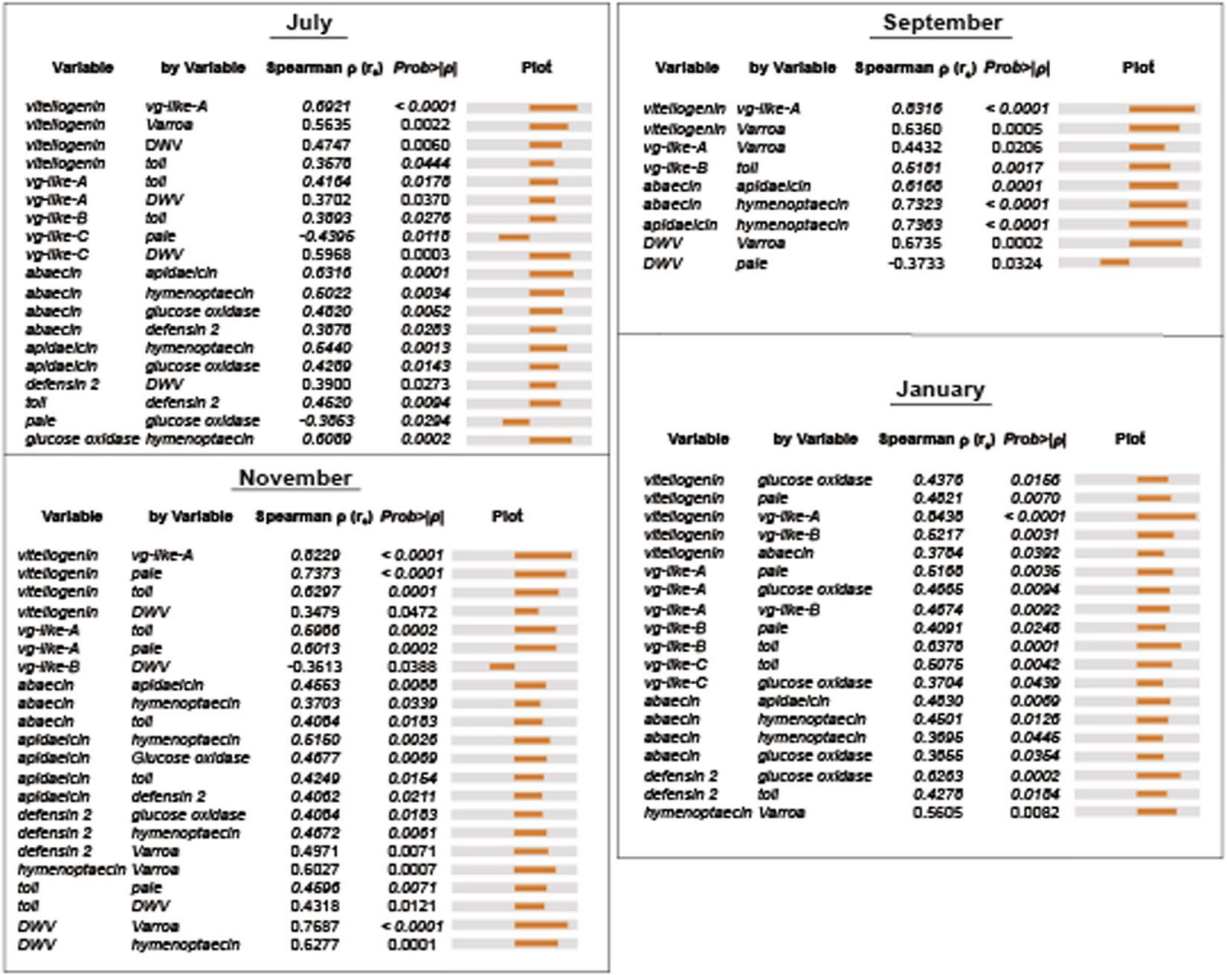
Spearman rank correlations for the different molecular markers that were determined significant (P < 0.05) at each evaluation period (July, September, November, and January; n = 26–32).

Molecular correlations emphasize physiological organization at the colony level and the relative strength of the relationships between nutritional markers (*vg* and *vg-like*), immunity, and pathogen levels (Fig. 7). Overall, *vg* expression was strongly correlated with *vg-like-A*. Expression of *vg-like-a*, *-b*, and *-c* correlated with immune expression depending on evaluation time point. In July, November, and January, *vg-like-a* was correlated with *toll*, which encodes a signal transducing protein involved in immune response and development. In November and January, *toll* was correlated with antimicrobial peptide gene expression. Transcript levels of the antimicrobial peptides were correlated with each other to varying degrees at each evaluation time point. DWV levels were correlated with colony *Varroa* infestation levels in September and November. *Vg* expression was correlated with *Varroa* infestation levels and DWV titers in July. In September, *vg* and *vg-like-a* expression were correlated with *Varroa* infestation levels.

## Discussion

Queen quality and nutrition are critical variables that influence honey bee colony performance and health. There are relatively few studies that quantify colony performance with respect to specific management practices on a large scale and we are unaware of studies that report fall through winter colony-level physiology in a warm southern climate. Our study evaluated the influence of queen age and supplemental pollen feeding at the apiary scale in the Imperial Valley, California. In addition to landscape dynamics, queen quality and age influence colony growth and productivity^51,53,54^. At two sites in November, colonies with young queens that were fed natural pollen showed significantly improved brood production relative to colonies with old queens that were fed artificial diet. At one site in January, colonies with young queens that were fed natural pollen outperformed colonies with old queens fed either diet. Overall, colonies that harbored young queens produced 24–25% more brood compared to colonies that harbored old queens in fall and winter. Queen reproductive potential is modulated by the rearing environment, aging, and possibly the extended microbiome^52,59–62^. In the context of this experimental design, our findings suggest that young queens could improve colony brood rearing and that supplemental feeding with natural pollen might further augment brood production, particularly during the fall-winter period when robust diutinus cohorts may be especially important for colony survival.

The increase in brood production from a feeding regime containing 25% natural pollen was not statistically significant despite trending that way in November and January. Limited pollen foraging and pollen stores were observed throughout the experiment, indicating that most hives had at least intermittent access to natural forage that may have diminished the impact of supplemental feeding with natural pollen. We also detected a significant interaction between feeding regimen and evaluation period. These results suggest that natural pollen likely provides essential/critical/important micronutrients or influences host gene expression, and the efficacy of artificial diets may be improved by modification according to spatial and temporal fluctuations in the nutritional landscape. Supplemental feed type did not influence gene expression, except for November transcript levels of *vg-like-A*, which were significantly increased in colonies fed natural pollen. Considering the role of *vg-like-A* in winter bee phenotypes, these results suggest that artificial feed containing natural diet components has the potential to improve colony health during overwintering, a period of elevated colony losses^63,64^.

Our findings revealed site-specific differences in pollen accumulation and brood production, which were recapitulated in nurse *vg* expression levels. These insights could be applied to commercial management by manipulating the size, location, or feeding regimen of apiaries with reduced colony-level *vg* expression to improve nutritional conditions at those sites.

The observed differences among apiaries suggest the importance of landscape variation in studies of colony health. Although we observed little indication of disease at any of the 3 sites, records of dysentery in the Imperial Valley indicate site-specific disease outbreaks (Bryan Ashurst pers. Comm.) that may in part explain differences by site, particularly differential immune gene expression.

The combined results indicate the production of diutinus bees in a warm southern climate as evidenced by a mean reduction in brood rearing and a net increase in nurse *vg* expression leading up to winter. Honey bee *vg* acts as a storage molecule and precursor to proteinaceous secretions produced by nurse bees to rear brood^65–67^. This ubiquitous glycolipoprotein also plays a crucial role in mediating the stresses incurred during overwintering. *Vg* has undergone significant past and ongoing stretches of adaptive evolution with a large proportion of amino acid changes fixed by positive selection^68^. The *vg* homologs *vg-like-A*, -*B*, and *-C* were recently identified in all sequenced hymenopteran genomes and may have arisen from ancient gene duplications of *vg*^69^. *Vg-like-A* shares close structural and functional similarities with *vg*^69^. We identified temporal expression patterns that were consistent with the role of *vg* and *vg-like-A* in life span regulation and winter bee phenotypes^10,14^, but *vg-like-A* exhibited a sizeable fold-change earlier in the season and a larger relative response throughout. Moreover, transcript levels of both *vg* and *vg-like-A* were influenced by site, but subtle differences in their responses over time suggest they may be differentially regulated. These differences suggest that *vg-like-A* shows promise as an additional colony-level biomarker that may provide complementary information to *vg* when studying colonies across a dynamic nutritional landscape. *Vg-like-B* responds to oxidative stress and is linked to over-wintering phenotypes but to a lesser extent than *vg-like-A*^58^. The function of *vg-like-C* is currently unknown but its expression patterns are consistent with functional divergence from *vg* and the other homologs. We provide the first report of colony-level expression of *vg-like* genes in the honey bee. We determined that the relative expression levels of *vg-like-a*, *-b*, and *-c* in pooled nurse samples were similar to those previously reported in individual bee fat bodies^58^ and ants^69^. Together with *vg*, these homologs show promise as prototypical biomarkers that correlate with nutritional and immunological status.

Immunity is intimately tied to nutritional status, and malnourished individuals are more susceptible to disease. In northern climates overwintering success relies on consistent cold temperatures that keep bees in the hive in a state of nutrient conservation. Although simple sugars are metabolized for heat, the proteins and lipids needed to rear brood in the spring are conserved. Consistent with this conservation hypothesis, overwintering in northern climates is associated with reduced immune gene expression at the colony level^2^. In contrast, we detected increased or stable immune expression, a significant energy investment for bees^70,71^. This fundamental difference suggests a colony level expenditure of proteins that may represent a net negative cost to colonies overwintering in southern climates. An additional related cost, pollen species diversity in the Imperial Valley is likely reduced compared to areas with greater average rainfall and less agricultural intensity^37^. Pollen species diversity is positively correlated with increased nutritional quality^6^. The continuous foraging effort we documented throughout the winter months likely results in the collection of incomplete pollen nutrition, compounding the cost associated with continuous immune expression.

## Conclusion

Here we provide the first comprehensive assessment of overwintering honey bee colonies in a southern climate. Our results detail a critical point in the bee life cycle with strong selection on colony survival. We recorded a 45% colony loss leading up to and concurrent with the seasonal production of diutinus bees. In northern climates, colonies typically revert to a broodless state. In contrast, we recorded no surviving colonies that were broodless, and activity outside the hive continued throughout the winter. Thus, colony behavior and physiology that accompany overwintering can take multiple forms depending on climate but retain signatures of honey bee life history traits as evidenced by group-level temporal expression patterns of *vitellogenin*.

Effective, high-throughput characterization of colony physiology can inform management decisions and land use practices that lead to improved honey bee health and performance^37,44,72^. To realize this goal on a commercial scale, colony-level molecular biomarkers require further development and characterization to identify the most sensitive and therefore useful targets. It is especially important to investigate their variation across a range of seasonal, geographic, and management contexts. Physiological measures of single adult individuals can fluctuate dramatically due to available forage, supplemental nutrition, seasonal colony demography, and pathogen loads^38^. Here we used a colony-level approach to integrate standard methods of hive evaluation with molecular measures of physiology. The honey bee colony is an adaptively organized entity that acquires and assimilates nutrition via age-based distribution of labor. To obtain molecular signal, we field-collected separate pools of foragers (n = 25) and nurses (n = 50) based on the robust spatial variation in worker tasks. We observed markedly higher *vg* expression in nurses compared to foragers, a well-established pattern based on studies using individual bees^11,73,74^. These differences verify the use of a pooled sampling approach to measure colony-level physiology in large field studies.

## Methods

### Experimental setup and colony management

In March 2016, two hundred and fifty two commercial colonies were established from splits of large, healthy parent colonies sourced from Ashurst Bee Co. Inc. (Westmoorland, CA), which managed all colonies in this study. Colonies were distributed across three sites (Supplementary Figs S12 and S13): Site 1 (Best 115 E & W, 33°01′′38.8′′N 115°31′07.8′′W), Site 2 (Young and River, 33°07′58.8′′N 115°34′05.3′′W), and Site 3 (Jacobsen 111, 33°04′30.5′′N 115°31′14.9′′W). Half of the colonies were requeened with a new Italian queen while half of the colonies retained an Italian queen from the same commercial breeding source that had spent a year in a commercially managed colony. At the start of colony evaluations in July 2016, these young queens were approximately 4 months old and old queens were approximately 16 months old. Colonies were also assigned feeding regimes consisting of patties made either entirely from artificial pollen substitute or from a mixture of substitute plus 25% dried natural pollen. This natural pollen consisted of a 1:1 (w/w) mix of a commercially available corbicular pollen source (Great Lakes Pollen Mix, Great Lakes Bee Supply, Galesburg, MI, USA) and almond pollen collected from pollen traps, vacuum sealed, and stored at −20 °C by USDA-ARS personnel. Feeding treatments were applied to colonies approximately every 21 days.

Colonies were evaluated in July, September, and November 2016, and in January 2017. Evaluations consisted of inspecting each frame and recording the area covered by sealed brood, open brood, and stored pollen. Frames were scored as 1/8, 1/4, 1/2, 3/4, or full (1) covered by each component by the same two researchers throughout the study. The researchers who performed frame scoring were calibrated against each other to reduce variance. These data were summed and expressed as frames of brood or pollen per colony. In January 2017, a total of 107 colonies had survived across all three sites and treatment combinations (see Supplementary Table S1 for details).

A representative subset of 12 colonies per site (36 total hives) were further sampled for molecular analyses at each time point. Pooled samples of two distinct groups of worker bees were collected based on the association between spatial variation in colony tasks and temporal polyethism^75–77^. Incoming forager and guard bees were collected by vacuum aspiration from hive entrances to represent a cohort of older workers^78^. Brood nest nurse bees were collected from the center of a healthy brood frame to represent a cohort of younger workers engaged in nutritional assimilation and brood rearing. All bees were sampled into 50 ml conical tubes, immediately frozen on dry ice, and stored at −80 °C for further processing (Supplementary Fig. S14).

### Nucleic acid extractions

Separate pools of 25 forager bees and 50 nurse bees were homogenized in lysis buffer (1.2 M guanidine thiocyanate, 0.6 M ammonium thiocyanate) using a rotary homogenizer at a volume of 0.5 ml lysis buffer per bee. One milliliter of each homogenate was added to a 2 ml bead-beating tube containing 0.2 g of 0.1 mm silica beads, immediately frozen on dry ice, and stored at −80 °C until nucleic acid extractions. Prior to extraction, the samples were thawed at 60 °C for 5 minutes, bead beaten for a total of 2 min in 30 s intervals and centrifuged to recover the supernatant. The RNA fraction was purified from 300 μl of the resulting supernatant using a GeneJet RNA Purification Kit (Thermo Fisher Scientific) according to the manufacture’s instructions. The DNA fraction was purified from 300 μl of the resulting supernatant using a GeneJet DNA Purification Kit (Thermo Fisher Scientific) according to the manufacturer’s instructions for Gram-positive bacteria.

### Gene expression analyses

*Vitellogenin* (*vg*), *vg-like-A*, *vg-like-B*, *and vg-like-C*, *abaecin*, *apidaecin*, *hymenoptaecin*, *defensin 2*, *glucose oxidase*, *toll*, and *pale* mRNA levels were measured by quantitative PCR (qPCR) and cDNA template generated from the purified RNA fraction of pooled bee samples. cDNA synthesis was carried out using a RevertAid First Strand cDNA Synthesis Kit (Thermo Fisher Scientific). PCR reactions were performed in triplicate as follows: initial denaturation at 95 °C for 5 minutes; 40 cycles with denaturation at 95 °C for 15 s; and a primer-pair-specific annealing and extension temperature (Supplementary Table S2) for 30 seconds. The reactions were carried out using iTaq^TM^ Universal SYBR® Green Supermix (Biorad) in triplicate on an CFX96^TM^ Real-Time PCR Detection System (Biorad). To confirm the absence of contaminating genomic DNA and primer dimers in the qPCR assay, we monitored amplification and melting curves in negative controls consisting of DNase-treated total RNA without reverse transcriptase. Relative gene expression was determined based on standardized Ct values (Δ Ct)^79^ using actin as a reference gene.

### Quantification of *Varroa*, deformed wing virus, and *Nosema ceranae* levels

The prevalence of infection by the microsporidian parasite *N*. *ceranae* was measured by qPCR using the DNA fraction of pooled bee samples as template. A standard curve was generated using serial dilutions of a plasmid standard containing a 104 base pair product of the *N*. *ceranae* ribosomal rRNA gene (Bourgeois *et al*., 2010). PCR reactions were performed using gene-specific primers (Online Resource 1 Table S1) as follows: initial denaturation at 95 °C for 5 minutes; 40 cycles with denaturation at 95 C for 15 s; and a combined annealing and extension step at 58 °C for 30 seconds. The reactions were carried out using iTaq^TM^ Universal SYBR® Green Supermix (Biorad) in triplicate on an CFX96^TM^ Real-Time PCR Detection System (Biorad). Sample C_q_ values were transformed using the linear equation obtained from a logarithmic standard curve of 2 × 10^3^ to 2 × 10^8^ copies of the *N*. *ceranae* rDNA template. Standard curve efficiencies ranged from 92–101% with R^2^ between 0.988–0.999. The qPCR results were expressed as the number of *N*. *ceranae* rRNA gene copies per bee after adjusting the values to account for sample volumes. Samples with copy amounts below the range of detection of the standard curve were assigned a value of 10^4^, corresponding to the lower limit of detection after adjustment for volume.

DWV titers were measured by qPCR using cDNA template generated from the purified RNA fraction of pooled bee homogenates^80,81^. Relative viral levels were determined based on standardized Ct values (Δ Ct)^79^ using DWV primers (Supplementary Table S1) and actin as a reference gene.

### Statistical analyses

All analyses were conducted in JMP v11 and Prism v7. Dependent variables were evaluated for normality using fit statistics and probability plots. Variables with deviations from normality were re-evaluated after transformation. The effects of queen age, supplemental feeding, and apiary site on brood production over time were analyzed by two-way repeated measures ANOVA and post-hoc comparisons were conducted using pairwise t-tests with Bonferroni adjustments. Pollen stores and treatment combination effects were analyzed at each site and evaluation period by Kruskal-Wallis (K-W) test and post hoc contrasts were conducted using pairwise Mann-Whitney-Wilcoxon tests with Bonferroni adjustments. The effects of apiary site and evaluation period on gene expression were analyzed by two-way ANOVA and post-hoc comparisons were conducted using pairwise t-tests with Bonferroni adjustments.

### Data availability

The datasets generated during and/or analysed during the current study are available from the corresponding authors on reasonable request.

## Electronic supplementary material

Supplementary information
