# Supplementary material for: Honey bees overwintering in a southern climate: longitudinal effects of nutrition and queen age on colony-level molecular physiology and performance
Source: Sci Rep. 2018 Jul 11;8:10475. doi: 10.1038/s41598-018-28732-z (PMC6041268; doi:10.1038/s41598-018-28732-z)
Supplement: Supplementary file 1 — Supplementary information [file 41598_2018_28732_MOESM1_ESM.docx]

Honey bees overwintering in a southern climate: longitudinal effects of nutrition and queen age on colony-level molecular physiology and performance

Vincent A. Ricigliano ^1^, Brendon M. Mott ^1^, Amy S. Floyd ^2^, Duan C. Copeland ^1,3^, Mark J. Carroll^1^, Kirk E. Anderson ^1,2^

^1^ USDA-ARS Carl Hayden Bee Research Center, Tucson, AZ 85719

^2^ Department of Entomology and Center for Insect Science, University of Arizona, Tucson, AZ 85721

^3^ Department of Microbiology, School of Animal & Comparative Biomedical Sciences; University of Arizona, Tucson, AZ, USA 85721

Corresponding author emails: [Vincent.Ricigliano@ARS.USDA.gov](mailto:Vincent.Ricigliano@ARS.USDA.gov) and Kirk.Anderson@ARS.USDA.GOV

**Figure S1.** Colony losses incurred throughout the course of the experiment (July 2016 – January 2017)


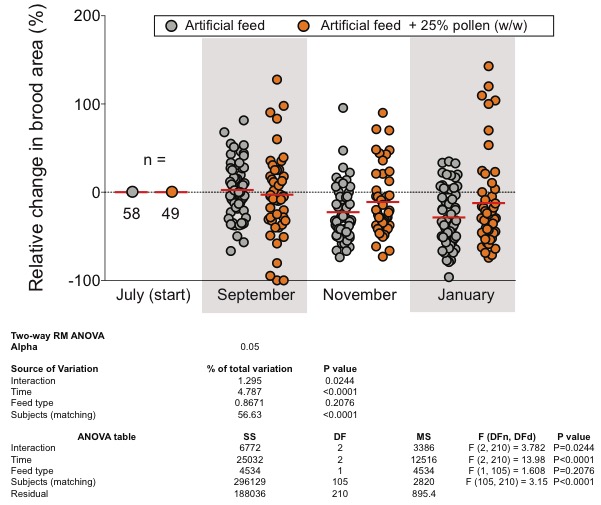


**Figure S2.** The effects of supplemental feeding on overall brood production. Values represent the percent change in total brood area relative to July. Red horizontal lines indicate the mean.


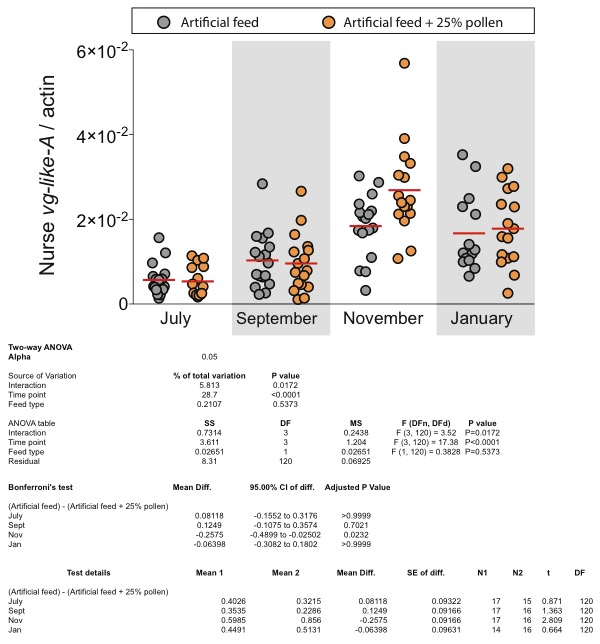


**Figure S3.** Expression of *vg-like-a* in nurse bees from colonies fed either artificial diet or artificial diet containing 25% natural pollen (w/w). Red horizontal lines indicate the mean. Asterisk indicates a statistically significant difference in November at α = 0.05.

**Figure S4.** Transcript levels *of vg-like-A*, *vg-like-B*, and *vg-like-C* by site. Red horizontal lines indicate the mean. Horizontal lines above columns indicate statistically significant differences at α = 0.05

**Figure S5.** The effects of apiary site and evaluation period on immune-related gene expression analyzed by two-way ANOVA.

**Figure S5 (continued).** The effects of apiary site and evaluation period on immune-related gene expression analyzed by two-way ANOVA.

**Figure S6.** Transcript levels of immune-related genes differentially expressed by site. Horizontal lines above columns indicate statistically significant differences at α = 0.05. Error bars represent standard error (SE).

**Figure S7.** The effects of apiary site and evaluation period on total brood production measured in frame coverage equivalents and analyzed by two-way repeated measures ANOVA. Error bars represent standard error (SE).

**Figure S8.** The effects of apiary site and evaluation period on capped and open brood production measured as frame coverage equivalents and analyzed by two-way repeated measures ANOVA

**Figure S9.** Relationship magnitudes of significant correlations concerning total brood production, pollen stores and relative nurse *vitellogenin* expression indicated by Spearman’s rho (ρ) values (scale from -1 to 1).

**Figure S10**. Relative deformed wing virus levels in pooled forager bees normalized to host actin and expressed on a log scale. Error bars represent standard error (SE) (n = 31.

**Figure S11.** Absolute quantification of *Nosema* 16S rDNA (rRNA gene) copies in pooled nurses expressed per bee. Error bars represent standard error (SE).

**Figure S12.** Apiary locations monitored in this study. Site 1 (Best 115 E & W, 33°01'38.8"N 115°31'07.8”W), site 2 (Young and River, 33°07'58.8"N 115°34'05.3"W), and site 3 (Jacobsen 111, 33°04'30.5"N 115°31'14.9”W. Imagery 2018 Google, Map data 2018 Google (United States)

**Figure S13.** Schematic representation of the experimental design used in this study.


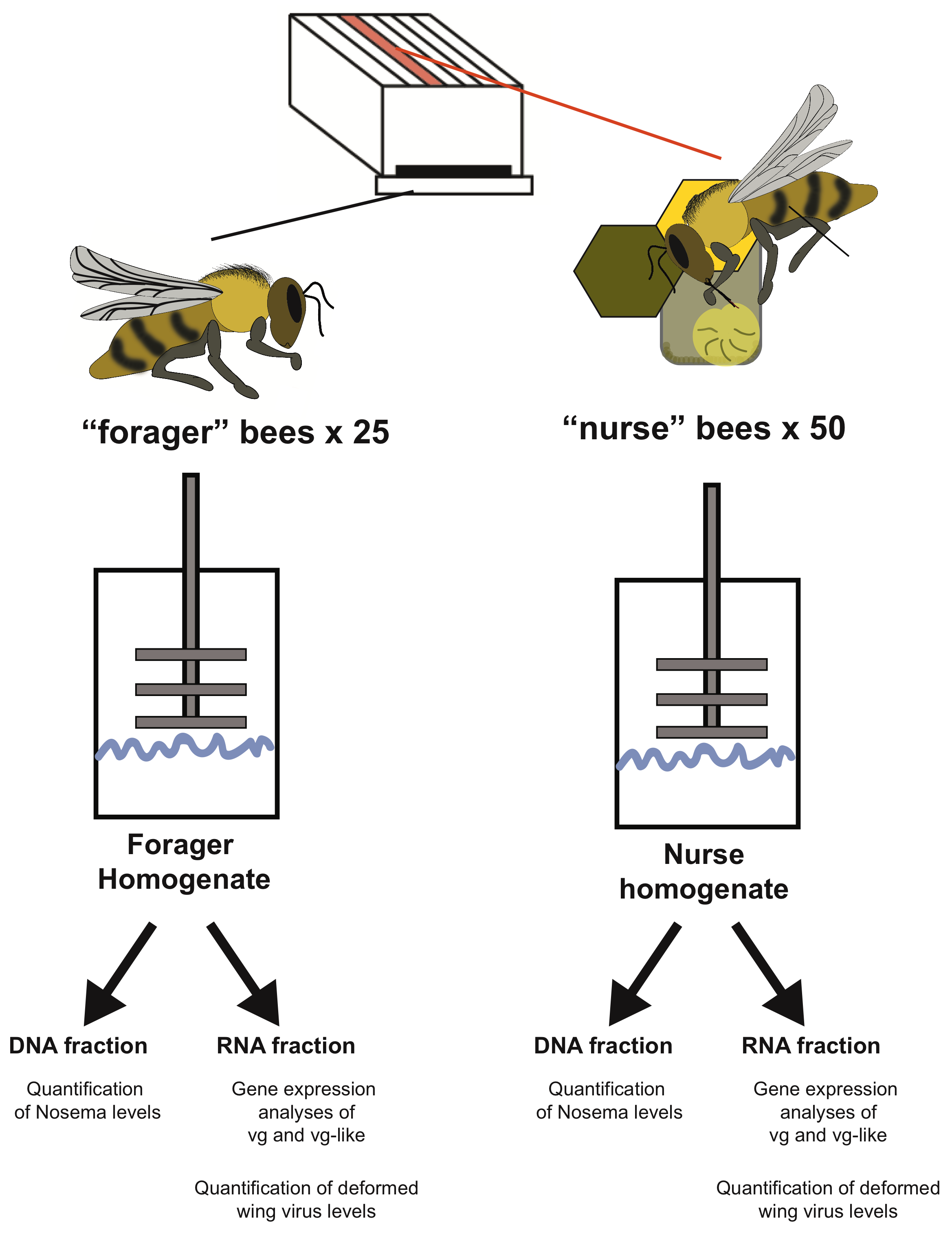


**Figure S14.** Processing schematic for field-collected honey bee samples. For each colony, pools of 50 “nurse” bees were collected from brood frames located in the center of the brood nest and pools of 25 incoming “forager” bees were collected from hive entrances.

**Table S1**. The number of surviving colonies representing each treatment combination at each site

| **Site** | **Young queen / artificial feed** | **Young queen / artificial feed +25% pollen** | **Old queen / artificial feed** | **Old queen / artificial feed +25% pollen** |
| --- | --- | --- | --- | --- |
| 1 | 9 | 3 | 8 | 11 |
| 2 | 14 | 9 | 11 | 9 |
| 3 | 6 | 11 | 10 | 6 |

**Table S2.** Primers used in this study

| **Gene**  **(accession number)** | **Forward 5’-3’** | **Reverse 5’-3’** | **Annealing temperature (°C)** | **Study** |
| --- | --- | --- | --- | --- |
| *Nosema ceranae* rRNA  (DQ486027) | AAGAGTGAGACCTATCAGCTAGTTG | CCGTCTCTCAGGCTCCTTCTC | 58.0 | Bourgeois et al., 2010 |
| *actin*  (XM_623378) | TGCCAACACTGTCCTTTCTG | AGAATTGACCCACCAATCCA | 55.0 | Alaux et al., 2011 |
| *vitellogenin (vg)*  (AJ517411) | GTTGGAGAGCAACATGCAGA | TCGATCCATTCCTTGATGGT | 57.5 | Salmela et al., 2016 |
| *vg-like-A* (XM_001121939.3) | GTTTATGACGAAAATGGACACCT | TGAACAGTTTCCTCGTGAGTT | 57.5 | Salmela et al., 2016 |
| *vg-like-B*  (XM_006561115.1) | ATTGCAACAATGCAGTTTGAATCAT | GCAAATCAGCCACATCTTTAGGA | 57.5 | Salmela et al., 2016 |
| *vg-like-C*  (XM_001122505.3) | AACGCGATCACATCAGTCGT | CGTGCCGCCAACAGATATGG | 55.0 | Salmela et al., 2016 |
| Deformed wing virus (DWV) | CAGTAGCTTGGGCGATTGTT | AGCTTCTGGAACGGCAGATA | 56.0 | Cox-Foster et al., 2007 |
| *abaecin*  (GB18323) | CAGCATTCGCATACGTACCA | GACCAGGAAACGTTGGAAAC | 55.0 | Evans et al., 2006 |
| *apidaecin*  *(*GB17782) | TAGTCGCGGTATTTGGGAAT | TTTCACGTGCTTCATATTCTTCA | 55.0 | Evans et al., 2006 |
| *defensin2*  *(*GB10036) | GCAACTACCGCCTTTACGTC | GGGTAACGTGCGACGTTTTA | 55.0 | Evans et al., 2006 |
| *hymenoptaecin*  *(*GB17538) | ctcttctgtgccgttgcata | GCGTCTCCTGTCATTCCATT | 55.0 | Evans et al., 2006 |
| *toll*  *(*GB18520) | TAGAGTGGCGCATTGTCAAG | ATCGCAATTTGTCCCAAAAC | 55.0 | Evans et al., 2006 |
| *glucose oxidase*  *(NM_001011574.1)* | GAGCGAGGTTTCGAATTGGA | GTCGTTCCCCCGAGATTCTT | 59.0 | Yang et al., 2005 |
| *pale*  *(NM_001011633.1)* | TACTTCGTCGCG GATAGCTT | TACGCGAACGAT GTCTTCAG | 60.0 | Richard et al., 2011 |
